# The effect of endothelial progenitor cell transplantation on neointimal hyperplasia and reendothelialisation after balloon catheter injury in rat carotid arteries

**DOI:** 10.1186/s13287-021-02135-w

**Published:** 2021-02-03

**Authors:** Wei Wang, Yingqian Zhang, Hui Hui, Wei Tong, Zechen Wei, Zhongxuan Li, Suhui Zhang, Xin Yang, Jie Tian, Yundai Chen

**Affiliations:** 1 Medical School of Chinese PLA, Chinese PLA General Hospital, Beijing, 100853 China; 2 Department of Cardiology, the Sixth Medical Centre, Chinese PLA General Hospital, Beijing, 100853 China; 3 CAS Key Laboratory of Molecular Imaging, Institute of Automation, Chinese Academy of Sciences, Beijing, 100190 China; 4 University of Chinese Academy of Sciences, Beijing, China; 5 Beijing Advanced Innovation Center for Big Data-Based Precision Medicine, School of Medicine, Beihang University, Beijing, 100083 China

**Keywords:** Endothelial progenitor cells, Reendothelialisation, Neointimal hyperplasia, Angioplasty, Light-sheet fluorescence microscopy

## Abstract

**Background:**

Reendothelialisation is the natural pathway that inhibits neointimal hyperplasia and in-stent restenosis. Circulating endothelial progenitor cells (EPCs) derived from bone marrow (BM) might contribute to endothelial repair. However, the temporal and spatial distributions of reendothelialisation and neointimal hyperplasia after EPC transplantation in injured arteries are currently unclear.

**Methods:**

A carotid balloon injury (BI) model was established in Sprague-Dawley rats, and PKH26-labelled BM-derived EPCs were transplanted after BI. The carotid arteries were harvested on the first, fourth, seventh, and 14th day post-injury and analysed via light-sheet fluorescence microscopy and pathological staining (*n* = 3). EPC and human umbilical vein endothelial cell culture supernatants were collected, and blood samples were collected before and after transplantation. The paracrine effects of VEGF, IGF-1, and TGF-β1 in cell culture supernatants and serum were analysed by enzyme-linked immunosorbent assay (*n* = 4).

**Results:**

Transplanted EPCs labelled with PKH26 were attached to the injured luminal surface the first day after BI. In the sham operation group, the transplanted EPCs did not adhere to the luminal surface. From the fourth day after BI, the mean fluorescence intensity of PKH26 decreased significantly. However, reendothelialisation and inhibition of neointimal hyperplasia were significantly promoted by transplanted EPCs. The degree of reendothelialisation of the EPC^7d^ and EPC^14d^ groups was higher than that of the BI^7d^ and BI^14d^ groups, and the difference in neointimal hyperplasia was observed between the EPC^14d^ and BI^14d^ groups. The number of endothelial cells on the luminal surface of the EPC^14d^ group was higher than that of the BI^14d^ group. The number of infiltrated macrophages in the injured artery decreased in the EPC transplanted groups.

**Conclusions:**

Transplanted EPCs had chemotactic enrichment and attached to the injured arterial luminal surface. Although decreasing significantly after the fourth day at the site of injury after transplantation, transplanted EPCs could still promote reendothelialisation and inhibit neointimal hyperplasia. The underlying mechanism is through paracrine cytokines and not differentiation into mature endothelial cells.

**Supplementary Information:**

The online version contains supplementary material available at 10.1186/s13287-021-02135-w.

## Background

Coronary artery disease (CAD) is the leading cause of disability and morbidity [1, 2]. Percutaneous coronary intervention has revolutionised the treatment of CAD, but it injures vascular endothelial cells (VECs). The injured vascular endothelium can cause vascular inflammation, which accelerates lipid deposition and thrombosis. These changes contribute to neointimal hyperplasia and in-stent restenosis (ISR) [3, 4]. The use of drug-eluting stents (DESs) inhibits neointimal hyperplasia but also inhibits reendothelialisation. ISR occurs in approximately 10% of patients with DESs [5]. Reendothelialisation is a natural pathway that inhibits neointimal hyperplasia and ISR. It is important for promoting reendothelialisation to prevent ISR. Endothelial progenitor cells (EPCs) may contribute to endothelial repair [6, 7]. EPCs capturing biomolecules are immobilised onto metal-based biomaterial surfaces to accelerate reendothelialisation [8]. Circulating EPCs may not only accelerate reendothelialisation by paracrine signalling but also differentiate into mature VECs [9, 10]. Alternatively, EPCs may never differentiate into mature VECs and only induce the proliferation and migration of original mature VECs nearby [11, 12]. EPCs could be divided into two main groups as follows to investigate their functions: haematopoietic and non-haematopoietic EPCs [13–16]. EPCs in the culture of mononuclear cells (MNCs) isolated from bone marrow (BM) after 7–14 days should be identified as haematopoietic EPCs. These spindle-shaped cells have pro-angiogenic paracrine actions [17–19]. EPCs in culture after 14–28 days should be identified as non-haematopoietic EPCs. These cells could replace and differentiate into mature VECs [20–22]. The early functional EPCs, a type of haematopoietic EPCs, are characterised by the expression of CD133, CD34, and vascular endothelial growth factor receptor 2 (VEGFR2) [23–26]. Although considerable efforts have been made to investigate how EPCs accelerate reendothelialisation, the underlying mechanism remains uncertain. The temporal and spatial distributions of reendothelialisation and neointimal hyperplasia of injured arteries after EPC transplantation are currently unclear.

We established a carotid balloon injury (BI) model and treated the BM-derived early EPCs. To fully evaluate temporal changes in neointimal hyperplasia and reendothelialisation after injury, the carotid arteries were harvested at different time points after transplantation. We present a three-dimensional (3D) investigation of the repair process after EPC transplantation for the purpose of locating and quantifying the extent of EPC attachment, reendothelialisation, and neointimal hyperplasia precisely. By evaluating the fluorescence intensity of PKH26-labelled EPCs at different time points after cell transplantation, we found that the attachment of EPCs to the damaged luminal surface did not persist. Transplanted EPCs were attached to the damaged luminal surface on the first day post-injury. From the fourth day, PKH26-labelled EPCs no longer appeared on the damaged surface. This indicates that EPCs do not directly differentiate into mature VECs but could still promote reendothelialisation and inhibit neointimal hyperplasia.

## Methods

### BM-derived EPC isolation and identification

**Fig. 1:**
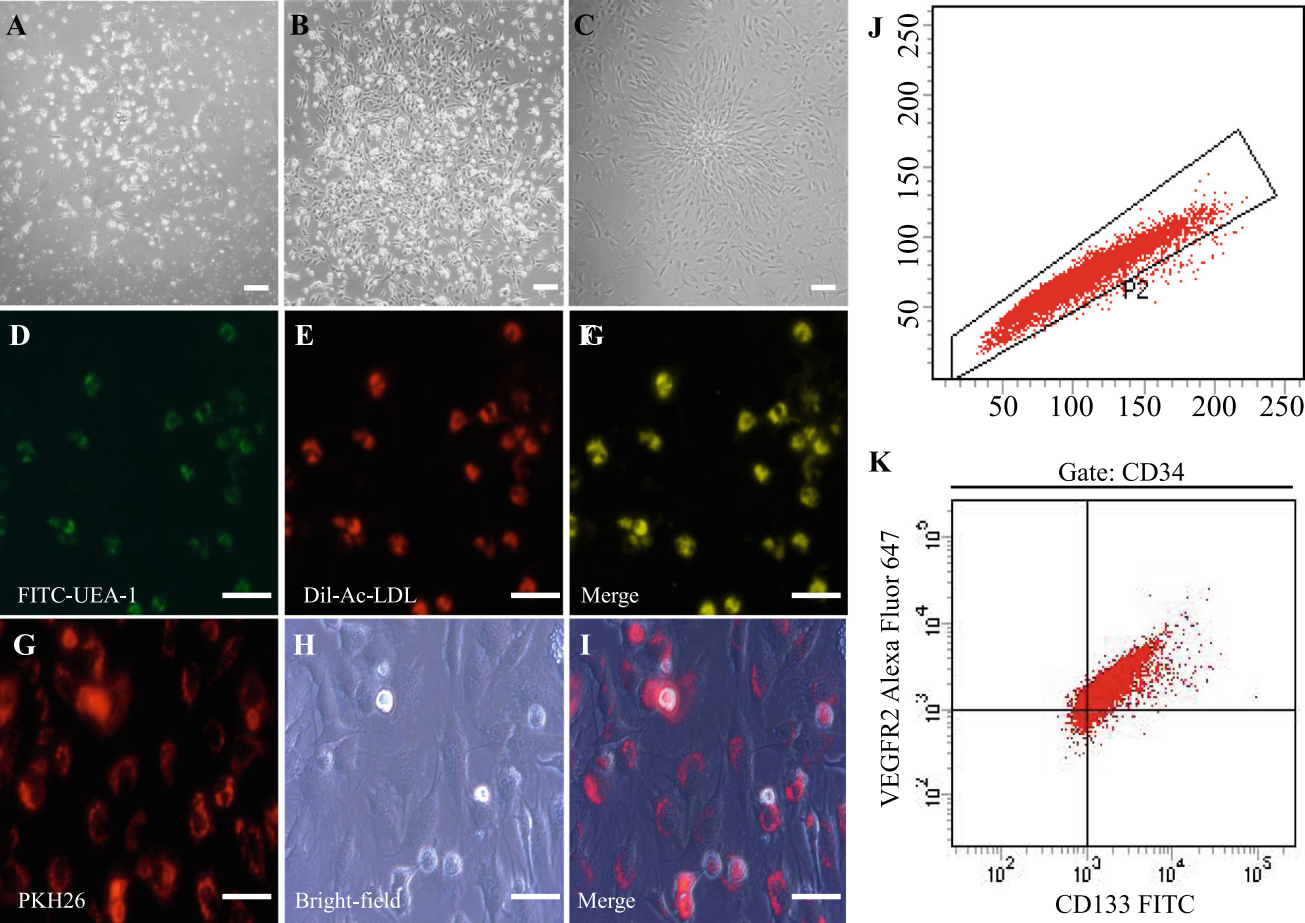
Identification of bone marrow-derived EPCs. **a** Adherent cells formed a cluster on the fifth day of culture. **b** The cell cluster grew into a colony on the seventh day of culture. **c** The typical colonies, consisting of a central core of rounded cells surrounded by spindle-shaped cells, appeared at the second day post-passage. Scale bar = 100 μm (**a**–**c**). Cells positive for FITC-UEA-1 staining (**d**, green) and Dil-ac-LDL staining (**e**, red). **f** Merged images represented double-positive for the uptake of Dil-ac-LDL and binding with FITC-UEA-1. The double-positive cells accounted for 85% of the total. Confocal microscopic images (**g**, red), bright-field microscopic images (**h**), and merged images of PKH26-labelled bone marrow-derived EPCs (**i**). Scale bar = 50 μm (**d**–**i**). **j** Cells were positive for CD34 in flow cytometry. **k** Cells were positive for CD34, VEGFR2, and CD133. The triple-positive cells accounted for 75% of the total

Sprague-Dawley (SD) rats (male, 140–150 g, SPF) from the Charles River Laboratories Supplier in China (Beijing, China, SCXK2016-0006) were euthanised by cervical dislocation. The rat tibias and femurs of both sides (four long bones from the hind limbs) were rinsed repeatedly to obtain the BM. To isolate MNCs, Histopaque-1083 (Sigma-Aldrich, St. Louis, MO, USA) was used for cell suspension by density gradient centrifugation. After isolation, MNCs were seeded on a 100-mm plate at a density of 2.5 × 10^6^ cells/cm^2^. These plates were coated at 37 °C using fibronectin (R&D Systems, Minneapolis, MN, USA) 24 h before seeding. Cells were cultured with EGM-2 (Lonza, Walkersville, MD, USA) containing 5% foetal bovine serum. After 24 h, floating cells in the medium were removed. The culture medium was changed daily for the first 3 days and then every 48 h. Five days later, cell clusters were observed. Spindle-shaped EPCs that appeared between the 7th and 14th days were identified as early EPCs, a type of haematopoietic EPC (Fig. 1a–c). Cells double-positive for Dil-ac-LDL and FITC-UEA-1 dual staining (85%) were identified as EPCs (Fig. 1d–f). PKH26-labelled EPCs were monitored for the fluorescent signal of PKH26 (Fig. 1g–i). Cells were triple-positive for VEGFR2, CD34, and CD133 (75%) in flow cytometry (Fig. 1j, k). EPCs at passage 3 were obtained and used in further experiments.

Between the 7th and 14th days of culture, cells were prepared as a single cell suspension. After resuspension in fluorescence-activated cell sorting buffer (100 μL), 5 × 10^5^ cells were incubated with rabbit anti-CD133/FITC conjugated antibody (1:100; Bioss Co., Ltd., Beijing, China, bs-4770R-FITC), CD34 monoclonal antibody (QBEND/10), PE (1:10; Thermo Fisher Scientific Inc., MA, USA, MA1-10205), and VEGFR2 (D5B1) rabbit mAb (Alexa Fluor® 647 conjugate) (1:50; Cell Signaling Technology Inc., MA, USA, 12658) for 60 min at 4 °C in the dark. The cells were washed twice with phosphate-buffered saline (PBS) (P3813, Sigma-Aldrich). After resuspension in fluorescence-activated cell sorting buffer (500 μL), cells were analysed by flow cytometry (BD LSRFortassa; Becton Dickinson, NJ, USA) with BD FACSDiva software (version 7.0).

During the 8 days of culture, attached EPCs were washed with PBS and subsequently stained with 1,1′-dioctadecyl-3,3,3′,3′-tetramethylindocarbocyanine-labelled acetylated low-density lipoprotein (Dil-ac-LDL, Maokang Biotechnogy Co., Ltd., Shanghai, China, MP6013) at a concentration of 10 mg/L. After incubation at 37 °C and 5% CO_2_ for 4 h, cells were fixed with 2% paraformaldehyde (PFA) in PBS for 10 min. Fluorescein isothiocyanate-labelled *Ulex europaeus* agglutinin (FITC-UEA-1, Maokang Biotechnogy Co., Ltd., Shanghai, China, MP6308) at a concentration of 10 mg/L was added. The cells were examined under a confocal fluorescent microscope (TCS SP5 II, Leica Microsystems, Germany).

### Animal protocols

**Fig. 2:**
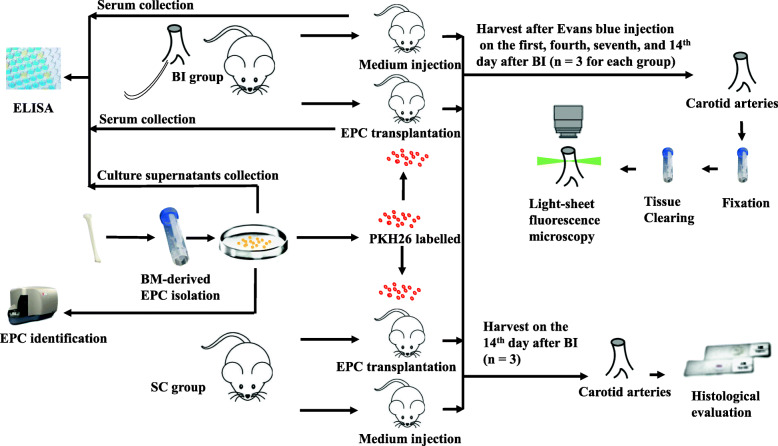
Flow chart of the protocol. Animal models of BI in carotid arteries received PKH26-labelled EPCs or medium and were euthanised at different time points. Carotid arteries were harvested and analysed via a light-sheet fluorescence microscope and pathological staining. Cell culture supernatants were collected, and blood samples were taken before and after transplantation for the paracrine function evaluation of the EPCs

All animals were housed in a 12-h light/dark cycle room at a controlled temperature (23 ± 2 °C) and humidity (50–60%) with free access to food and water. The final sample size was determined based on the preliminary results. SD rats (weight, 250–300 g; age, 7 to 8 weeks; male; SPF) were obtained from the Charles River Laboratories Supplier in China (Beijing, China, SCXK2016-0006). All rats were fed regular rodent chow for the first week. The rats were anesthetised with 40 mg/kg body weight pentobarbital via intraperitoneal injection and subjected to BI to the right carotid artery. Briefly, the rats were placed in the supine position and the bifurcation of the right carotid artery was exposed via a midline neck incision. A balloon angioplasty catheter was inserted through the incision in the artery. The balloon was then inflated at 2–3 atm and passed through the artery three times. The punched area was sealed, and the common carotid artery resumed blood flow. Rats received either 1 × 10^6^ PKH26-labelled EPCs or EGM-2 medium via intravenous tail vein injection after injury. PKH26-labelled EPCs injected via the tail vein after BI could be monitored (Fig. 1g–i) for the fluorescent signal of PKH26. Ibuprofen (15 mg/kg/day) was provided ad libitum as a pain killer for 3 days after the procedure. On the first, fourth, seventh, and 14th days after BI, rats were euthanised and carotid arteries were harvested (Fig. 2).

Rats were divided into the nine following groups: EPC^1d^ group (*n* = 3), EPC transplantation after BI and harvested on the first day; BI^1d^ group (*n* = 3), medium injection after BI and harvested on the first day; EPC^4d^ group (*n* = 3), EPC transplantation after BI and harvested on the fourth day; BI^4d^ group (*n* = 3), medium injection after BI and harvested on the fourth day; EPC^7d^ group (*n* = 3), EPC transplantation after BI and harvested on the seventh day; BI^7d^ group (*n* = 3), medium injection after BI and harvested on the seventh day; EPC^14d^ group (*n* = 6), EPC transplantation after BI and harvested at the 14th day; BI^14d^ group (*n* = 6), medium injection after BI and harvested on the 14th day; and sham operation control (SC) group (*n* = 30).

### Perfusion and tissue preparation

Rats were anesthetised with 40 mg/kg body weight pentobarbital via intraperitoneal injection and received 10 mg/kg body weight 0.1% Evans blue dye (SL7202, Coolaber) by intravenous tail vein injection 30 min before tissue harvesting. The animals were perfused transcardially with 0.01 M PBS (P3813, Sigma-Aldrich) and 4% PFA (158,127, Sigma-Aldrich) in PBS. The common carotid arteries were harvested and fixed in 4% PFA at 4 °C for at least 24 h before tissue clearing.

### Tissue clearing procedure

The common carotid artery embedded in 1% agar was dehydrated using the gradient dehydration method. Benzyl alcohol and benzyl benzoate (1:2) were used as the tissue clearing solutions. All steps were performed at 18 °C. Finally, the sample was placed in the dark overnight at 4 °C.

### Light-sheet fluorescence microscopy

All carotid arteries were collected from the origin of the common carotid artery to the bifurcation. The volumes of the neointima of different arteries were normalised by dividing by the longest carotid artery and multiplying by their lengths. Three-dimensional images of cleared carotid arteries were acquired using a light-sheet fluorescence microscope (Ultramicroscope II, LaVision Biotec, Bielefeld, Germany). We used a × 2.5 objective lens (Mv PLAPO 2VC, Olympus) or a × 4 objective lens (Mv PLAPO 2VC, Olympus) covered with a 6-mm working distance dipping cap. The laser source was a white light supercontinuum laser (SuperK EXTREME, NKT Photonics, Cologne, Germany). To observe cell distribution and artery morphology, the filters were set as 551/40 nm excitation and 567/50 nm emission for PKH26 and 640/30 nm excitation and 690/50 nm emission for Evans blue. The step size, scanning range, and exposure time were set to 5 μm, 1 mm, and 100 ms, respectively. 3D projections of the tagged image file format images of the artery were obtained using Imaris software (Bitplane, Oxford Instruments Company).

### Image processing

Images from light-sheet fluorescence microscopy (LSFM) were stored as 16-bit tag image file format. First, all images were converted to 8-bit to normalise fluorescence intensity ranging from 0 to 255. Second, the maximum fluorescence intensity was chosen from the images of SC group as the threshold for segmenting PKH26-labelled EPCs and Evans blue-stained endothelium in experimental groups. The fluorescence intensities of all images were finally normalised from 0 to 1 range for statistical analysis. Images of haematoxylin and eosin (H&E) and immunofluorescence were analysed using ImageJ software (version 1.52a, Wayne Rasband, National Institutes of Health, USA).

### Morphometric analysis

Carotid arteries were trimmed and paraffin-embedded at the Histology and Comparative Pathology Facility. Tissue sections of 5 μm thickness from three different regions of the artery were collected. Arterial sections were stained with H&E. Endothelial cells were detected by the immunofluorescence staining of rabbit anti-CD31 antibody (Abcam, ab24590, 1:50 dilution). Inflammation was detected by immunofluorescence staining of mouse anti-CD68 antibody (Abcam, ab31630, 1:200 dilution). All images of arterial sections were acquired using a confocal microscope (Leica DM3000, Germany) with exposure time set as 500 ms.

### Analysis of EPC paracrine function

The paracrine function of EPCs was evaluated by determining the levels of vascular endothelial growth factor (VEGF), transforming growth factor-β1 (TGF-β1), and insulin-like growth factor-1 (IGF-1) in cell culture supernatants and serum of BI^1d^, EPC^1d^, and SC groups using enzyme-linked immunosorbent assay (ELISA) kits. Next, 1 × 10^6^ EPCs or human umbilical vein endothelial cells (HUVECs) were incubated in serum-free culture medium for 24 h. After collection and centrifugation, the cell culture supernatants and serum were stored at − 80 °C.

### Statistical analysis

Continuous variables consistent with a normal distribution are presented as mean ± SEM. Multiple group comparisons were performed using a one-way ANOVA followed by post hoc analysis using the least significant difference (LSD) *t* test or Dunnett’s T3 post hoc test using Welch’s ANOVA. Statistical comparisons were performed using Student’s *t* test for experiments consisting of two independent groups only. Two-sided tests were used throughout the experiment. *P* < 0.05 was considered statistically significant. Statistical analysis was performed using SPSS 19.0 (IBM, Armonk, NY, USA).

## Results

### The effect of EPC transplantation on neointimal hyperplasia in BI carotid arteries

**Fig. 3:**
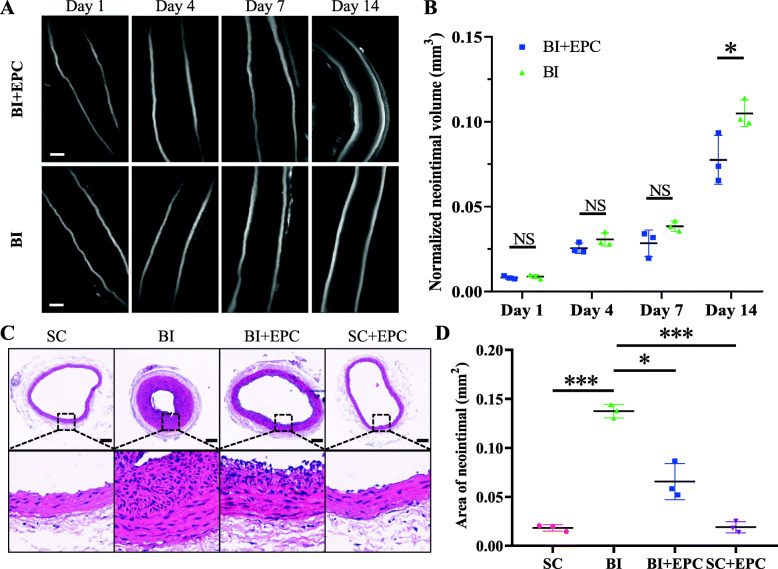
EPC transplantation attenuates neointimal hyperplasia after BI. **a** Autofluorescence of neointimal hyperplasia on the optical sections from each group at different time points. Scale bar = 250 μm. **b** Normalised volume of carotid artery of different groups on the first, fourth, seventh, and 14th day after BI (*n* = 3 for each group). **c** Cross-sections of carotid arteries stained with H&E in different groups on the 14th day after BI. **d** Neointimal area of different groups (*n* = 3 for each group). Data are presented as mean ± SEM (**b**, **d**) and were analysed by independent Student’s *t* test in **b** and by Welch’s ANOVA with Dunnett’s T3 post hoc test in **d**. SC, sham operation control; BI, balloon injury; BI+EPC, EPC transplantation after BI; SC+EPC, EPC transplantation after sham operation. NS, no statistical difference; *P < 0.05, **P < 0.01, ***P < 0.001 vs. the BI group

The neointimal volume increased from BI^1d^, BI^4d^, BI^7d^, to BI^14d^. EPCs were transplanted immediately after BI and the volume of the neointima increased from EPC^1d^, EPC^4d^, EPC^7d^, to EPC^14d^. Compared with BI^14d^ (0.105 ± 0.008 mm^3^), the neointimal volume of EPC^14d^ (0.078 ± 0.014 mm^3^) was significantly smaller (*P* = 0.044). There was no significant difference between EPC^1d^ and BI^1d^ (0.008 ± 0.001 mm^3^ vs. 0.009 ± 0.001 mm^3^, *P* = 0.552), EPC^4d^ and BI^4d^ (0.026 ± 0.003 mm^3^ vs. 0.031 ± 0.004 mm^3^, *P* = 0.145), and EPC^7d^ and BI^7d^ (0.028 ± 0.008 mm^3^ vs. 0.038 ± 0.003 mm^3^, *P* = 0.106), respectively. We also compared the neointimal area between the EPC^14d^ and BI^14d^ groups via H&E staining. The neointimal area in the EPC^14d^ group was smaller than that in the BI^14d^ group (0.066 ± 0.018 mm^2^ vs. 0.137 ± 0.008 mm^2^, *P* = 0.040) (Fig. 3).

### 3D distribution of transplanted EPCs and the effect of EPC transplantation on reendothelialisation

**Fig. 4:**
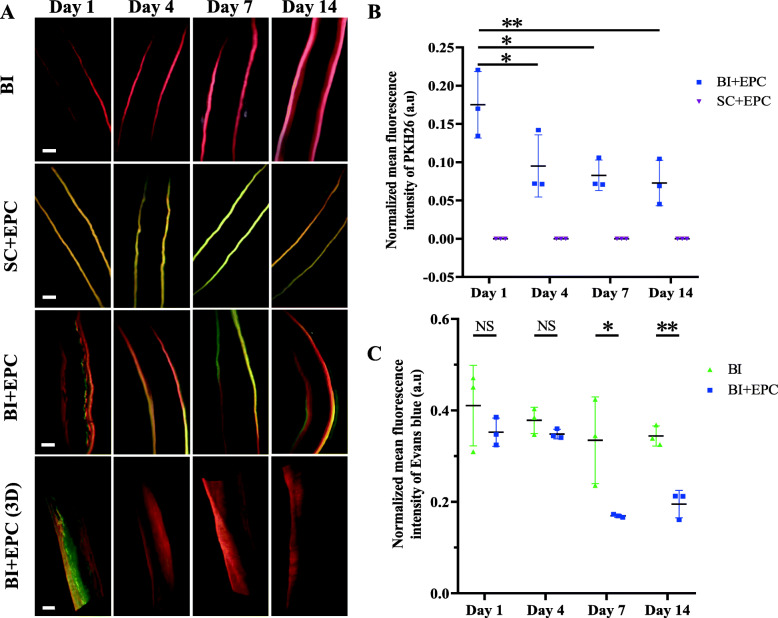
Distribution of transplanted EPCs and injured endothelium in the carotid artery. **a** Optical sections and 3D projections of the carotid arteries. Scale bar = 500 μm. **b** The normalised mean fluorescence intensity of PKH26 from transplanted EPCs at different time points (*n* = 3). **c** The normalised mean fluorescence intensity of Evans blue from the injured endothelium at different time points. Data are shown as mean ± SEM. Data were analysed by one-way ANOVA with LSD post hoc comparisons in **b** and were analysed by independent Student’s *t* test in **c**. NS, no statistical difference; *P < 0.05, **P < 0.01, ***P < 0.001. BI, balloon injury; BI+EPC, EPC transplantation after BI; SC+EPC, EPC transplantation after sham operation

We aimed to determine the reendothelialisation after BI using 3D evaluation. The distribution of transplanted EPCs changes in 3D over time, but the impairment of endothelial integrity was typically measured with cross-sections or longitudinal sections and subsequent histological analysis [27–29]. Transplanted EPCs labelled with PKH26 could be observed in the 551-nm channel under a light-sheet microscope and attached to the luminal surface of BI arteries on the first day. No PKH26-labelled cells were attached to the uninjured arteries. On the fourth day, the fluorescence intensity of PKH26 fluorescence decreased significantly on the luminal surface of the BI arteries. The normalised fluorescence intensity of PKH26 was significantly higher on EPC^1d^ than on EPC^4d^ (0.175 ± 0.434 vs. 0.095 ± 0.041, *P* = 0.023), EPC^7d^ (0.175 ± 0.434 vs. 0.083 ± 0.020, *P* = 0.012), and EPC^14d^ (0.175 ± 0.434 vs. 0.073 ± 0.030, *P* = 0.007) groups. Evans blue could penetrate the areas where the endothelium was permeable and stain the injured surface blue. The injured endothelium labelled by Evans blue was observed in the 611-nm channel under LSFM. The normalised fluorescence intensity of Evans blue decreased from BI^1d^ (0.411 ± 0.088) to BI^14d^ (0.344 ± 0.022), and from EPC^1d^ (0.352 ± 0.031) to EPC^14d^ (0.195 ± 0.030) groups. The normalised fluorescence intensity of Evans blue was significantly higher on BI^14d^ than on EPC^14d^ (0.344 ± 0.022 vs. 0.195 ± 0.030, *P* = 0.002), and the fluorescence intensity was higher on BI^7d^ than on EPC^7d^ (0.335 ± 0.095 vs. 0.169 ± 0.003, *P* = 0.039). However, there was no difference between BI^1d^ and EPC^1d^ (0.411 ± 0.088 vs. 0.352 ± 0.031, *P* = 0.340), or between BI^4d^ and EPC^4d^ (0.378 ± 0.029 vs. 0.348 ± 0.010, *P* = 0.163) groups (Fig. 4 and Additional file 1).

**Fig. 5:**
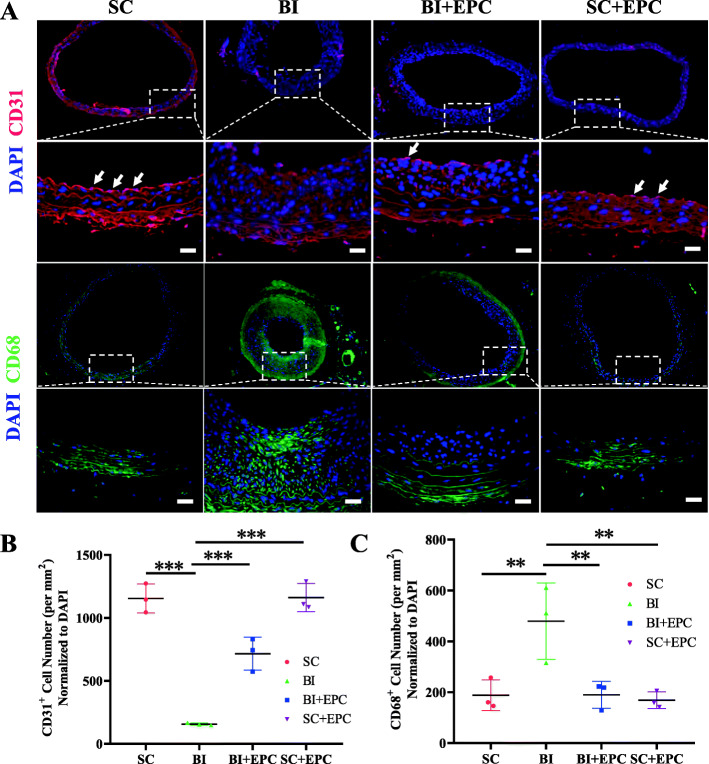
Immunofluorescence staining analysis. **a** Immunofluorescence of CD31 and CD68 in the carotid arteries of different groups. Arrows indicate endothelial cells. Quantification of CD31 (**b**) and of CD68 (**c**) staining from images in **a**. Data are presented as mean ± SEM and were analysed by one-way ANOVA with LSD post hoc comparisons, *n* = 3. NS, no statistical difference; *P < 0.05, **P < 0.01, ***P < 0.001 vs. the BI group. SC, sham operation control; BI, balloon injury; BI+EPC, EPC transplantation after BI; SC+EPC, EPC transplantation after sham operation

We also compared CD31 positive cells on the luminal surface between the EPC^14d^ and BI^14d^ groups via immunofluorescence. CD31 positive cell levels in EPC^14d^ were higher than those of BI^14d^ (716 ± 131 cells/mm^2^ vs. 156 ± 10 cells/mm^2^, *P* < 0.001), which is consistent with the results of the 3D evaluation. We evaluated the degree of macrophage infiltration in the right common carotid arteries between the BI^14d^ and EPC^14d^ groups via immunofluorescence. There were fewer CD68 positive cells in EPC^14d^ than in BI^14d^ (190 ± 53 cells/mm^2^ vs. 479 ± 150 cells/mm^2^, *P* = 0.004) (Fig. 5).

**Fig. 6:**
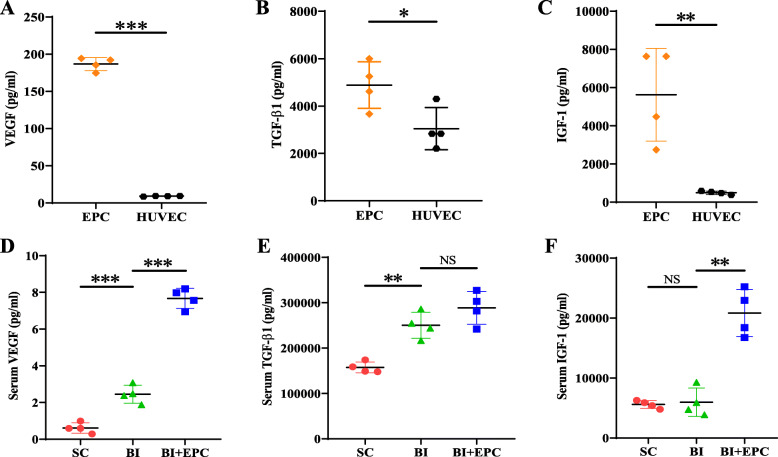
VEGF, TGF-β1, and IGF-1 in cell culture supernatants and the serum of EPC^1d^, BI^1d^, and SC groups. **a**–**c** The levels of VEGF, TGF-β1, and IGF-1 in EPC and HUVEC culture supernatants. Data are presented as mean ± SEM and were analysed by independent Student’s *t* test, *n* = 4. NS, no statistical difference; *P < 0.05, **P < 0.01, ***P < 0.001 vs. the HUVEC group. **d**–**f** The sera of EPC^1d^, BI^1d^, and SC were obtained. The levels of VEGF, TGF-β1, and IGF-1 in the sera were compared. Data are presented as mean ± SEM and were analysed by one-way ANOVA with LSD post hoc comparisons or Welch’s ANOVA with Dunnett’s T3 post hoc test, *n* = 4. NS, no statistical difference; *P < 0.05, **P < 0.01, ***P < 0.001 vs. the BI group. BI, balloon injury; EPC+BI, EPC transplantation after BI

The paracrine function of the EPCs was evaluated by measuring the levels of VEGF, TGF-β1, and IGF-1 in cell culture supernatants and the serum of BI^1d^, EPC^1d^, and the SC groups using ELISA kits. First, 1 × 10^6^ EPCs or HUVECs were incubated in serum-free culture medium for 24 h. In vitro, VEGF (186.700 ± 8.891 pg/mL vs. 9.073 ± 0.493 pg/mL, *P* < 0.001), TGF-β1 (4884.625 ± 986.662 pg/mL vs. 3041.499 ± 890.529 pg/mL, *P* = 0.032), and IGF-1 (5626.850 ± 2430.089 pg/mL vs. 494.274 ± 92.002 pg/mL, *P* = 0.006) levels of EPC culture supernatants were higher than those of HUVEC culture supernatants. The serum VEGF levels of the EPC^1d^ group were higher than those in the serum of the BI^1d^ group (7.673 ± 0.546 pg/mL vs. 2.455 ± 0.492 pg/mL, *P* < 0.001). The serum levels of IGF-1 in the EPC^1d^ group were higher than those in the serum of BI^1d^ (20,829.553 ± 3925.356 pg/mL vs. 5953.253 ± 2364.436 pg/mL, *P* = 0.004). There was no difference in the levels of TGF-β1 between EPC^1d^ and BI^1d^ (288,567.810 ± 36,198.920 pg/mL vs. 250,389.093 ± 28,614.938 pg/mL, *P* = 0.082) (Fig. 6).

## Discussion

This study demonstrated that transplanted EPCs have chemotactic enrichment and can be attached to the injured luminal surface. Although the mean fluorescence intensity of PKH26 decreased significantly on the luminal surface from the fourth day after transplantation, reendothelialisation was significantly accelerated and neointimal hyperplasia was inhibited in the EPC transplanted groups compared with the medium injection groups. An in vitro study found that EPCs could secrete VEGF, TGF-β1, and IGF-1. An in vivo study found that VEGF and IGF-1 levels increased significantly in the serum of EPC^1d^ compared with that in the BI^1d^ group. There was a trend of increasing TGF-β1 in the serum of the EPC^1d^ group. The possible mechanism by which EPCs facilitate reendothelialisation of the BI arteries is at least partially mediated by paracrine cytokines.

We established a rat carotid BI model to induce neointimal hyperplasia [30–32]. The mechanism of injury was similar to that of percutaneous transluminal coronary angioplasty. Neointimal formation and reendothelialisation are 3D processes. However, they are typically measured in two dimensions without an accurate reconstruction [33, 34]. Traditional two-dimensional analyses cannot fully evaluate the spatial distribution of neointimal hyperplasia and reendothelialisation. Selection bias as well as underestimation or overestimation will occur. For irregular geometry, the processes of neointimal hyperplasia and reendothelialisation should be evaluated in 3D. LSFM has been used to process 3D reconstruction of cardiac structures in zebrafish and mice [35, 36]. In combination with tissue clearing techniques, LSFM has been used to characterise the murine brain, cochleae, and atherosclerosis plaques [37–39]. Therefore, LSFM has ability to visualise the spatial distribution of endothelium injury, neointimal hyperplasia, reendothelialisation, and transplanted EPC distribution in BI arteries.

In this study, we applied the tissue clearing method in combination with LSFM for evaluation of intact neointimal in 3D. We found that the degree of neointimal hyperplasia changed with time after BI. The neointimal volume increased from BI^1d^, BI^4d^, BI^7d^, to BI^14d^. With EPC transplantation, neointimal volume also increased from EPC^1d^, EPC^4d^, EPC^7d^, to EPC^14d^. The restoration of the intact endothelium after injury is of great significance for the prevention of neointimal hyperplasia and stent thrombosis [40, 41]. Reendothelialisation can be accelerated by EPC transplantation [42–44]. The neointimal volume of EPC^14d^ was significantly smaller than that of BI^14d^, while there was no significant difference between EPC^1d^ and BI^1d^, EPC^4d^ and BI^4d^, or EPC^7d^ and BI^7d^.

When exploring the mechanisms by which transplanted EPCs accelerate reendothelialisation, previous studies have come to different conclusions. Hagensen et al. suggested that the migration of adjacent arterial endothelial cells is the only source of reendothelialisation [11, 12]. However, other studies have stated that transplanting EPCs not only promoted reendothelialisation through the paracrine function, but also differentiated into mature ECs [10, 45, 46]. Transplanted EPCs labelled with PKH26 were attached to the injured luminal surface on the first day after BI. For the undamaged arteries, the transplanted EPCs never adhered to the luminal surface. Our results support the idea that transplanted EPCs have chemotactic enrichment and adhere to the site of endothelial injury. The mechanism by which EPCs can migrate to the injured luminal surface has been revealed in previous studies. CXC chemokine receptor 4 positively increased CXCL12 production and promoted the chemotactic enrichment of EPCs together with CXCL12. MicroRNA-126 enriched in apoptotic VECs during endothelium injury increased the content of CXCL12 and promoted the migration of EPCs to the injured site [47, 48]. In this study, from the fourth day after BI, the mean fluorescence intensity of PKH26 decreased significantly. PKH26-labelled cells were monitored for 100 days and could be positive on the newborn cell membrane with cell proliferation. PKH26 did not appear in the reendothelialisation region from the fourth day after BI, indicating that the PKH26-labelled EPCs did not differentiate into mature endothelial cells to cover the injured luminal surface. Although transplanted EPC levels decreased significantly at the site of injury on the fourth day after transplantation, reendothelialisation at the site of injury and inhibition of neointimal hyperplasia were significantly promoted. Reendothelialisation of EPC^7d^ and EPC^14d^ groups was significantly better than that of the BI^7d^ and BI^14d^ groups, and a statistical difference in neointimal hyperplasia was observed between EPC^14d^ and BI^14d^. The number of endothelial cells on the luminal surface of EPC^14d^ was greater than that of BI^14d^. Infiltrated macrophage levels at the injured site decreased after EPC transplantation on the 14th day after BI. Therefore, the underlying mechanism of reendothelialisation promoted by EPC transplantation is not differentiation into mature ECs.

Active components of EPC paracrine secretion facilitated the proliferation and migration of VECs, and these effects were associated with the expression of VEGF and IGF [49–51]. VEGF can promote reendothelialisation, inhibit neointimal hyperplasia, and play a crucial role in vascular repair [45]. Levels of cytokines, such as VEGF, TGF-β1, and IGF-1, all increased after EPC transplantation [52, 53]. EPCs secreted VEGF, TGF-β1, and IGF-1 in vitro. VEGF and IGF-1 levels increased significantly in the serum of EPC^1d^ compared with that in the BI^1d^ group. There was a trend of increasing TGF-β1 in the serum of the EPC^1d^ group, but there was no significant difference compared with the BI^1d^ group. This could be associated with the serum being collected from a systemic blood sample instead of being obtained directly from the BI lesion. Therefore, the mechanism by which EPCs can facilitate reendothelialisation of the BI arteries is more likely to be paracrine cytokines. Moreover, previous studies selected the seventh, 14th, and 28th days after injury as the time points to study reendothelialisation. The changes in EPCs and reendothelialisation in the early period after BI and EPC transplantation, especially the time before the seventh day, are seldom investigated. Our study demonstrated that EPCs chemotactically adhere to the injured site and secrete cytokines in the early period of BI to promote reendothelialisation.

There are limitations to the present study. First, we used only a segment of the right common carotid artery instead of the entire artery due to the size requirements of LSFM; thus, the entire common carotid artery will be analysed in a future study. Second, our methodology requires the harvesting of the carotid artery of interest and thus does not allow sequential assessments in the same animal.

## Conclusions

We found that EPC transplantation attenuates neointimal hyperplasia and accelerates reendothelialisation after BI. Transplanted EPCs chemotactically adhere to the injured luminal surface and secrete cytokines in the early period. EPC transplantation promotes reendothelialisation through paracrine cytokines, such as VEGF, TGF-β1, and IGF-1, but not differentiation into mature endothelial cells.

## Supplementary Information

**Additional file 1:** **Movie S1.** 3D reconstruction of carotid arteries.

## Data Availability

All data generated or analysed during this study are included in this published article and its supplementary information files.

## Abbreviations

CAD: Coronary artery disease
VEC: Vascular endothelial cell
DES: Drug-eluting stent
EPC: Endothelial progenitor cell
MNC: Mononuclear cell
BM: Bone marrow
VEGFR: Vascular endothelial growth factor receptor
BI: Balloon injury
3D: Three-dimensional
SC: Sham operation control
SD: Sprague-Dawley
PBS: Phosphate-buffered saline
Dil-ac-LDL: 1,1′-Dioctadecyl-3,3,3′,3′-tetramethylindocarbocyanine-labelled acetylated low-density lipoprotein
PFA: Paraformaldehyde
FITC-UEA-1: Fluorescein isothiocyanate-labelled *Ulex europaeus* agglutinin
H&E: Haematoxylin and eosin
VEGF: Vascular endothelial growth factor
TGF: Transforming growth factor
IGF-1: Insulin-like growth factor
ELISA: Enzyme-linked immunosorbent assay
HUVEC: Human umbilical vein endothelial cell
LSD: Least significant difference
LSFM: Light-sheet fluorescence microscopy
